# Direction-of-Arrival Estimation with Coarray ESPRIT for Coprime Array

**DOI:** 10.3390/s17081779

**Published:** 2017-08-03

**Authors:** Chengwei Zhou, Jinfang Zhou

**Affiliations:** College of Information Science and Electronic Engineering, Zhejiang University, Hangzhou 310027, China; zhouchw@zju.edu.cn

**Keywords:** coprime array, DOA estimation, ESPRIT, off-grid, virtual sensor

## Abstract

A coprime array is capable of achieving more degrees-of-freedom for direction-of-arrival (DOA) estimation than a uniform linear array when utilizing the same number of sensors. However, existing algorithms exploiting coprime array usually adopt predefined spatial sampling grids for optimization problem design or include spectrum peak search process for DOA estimation, resulting in the contradiction between estimation performance and computational complexity. To address this problem, we introduce the Estimation of Signal Parameters via Rotational Invariance Techniques (ESPRIT) to the coprime coarray domain, and propose a novel coarray ESPRIT-based DOA estimation algorithm to efficiently retrieve the off-grid DOAs. Specifically, the coprime coarray statistics are derived according to the received signals from a coprime array to ensure the degrees-of-freedom (DOF) superiority, where a pair of shift invariant uniform linear subarrays is extracted. The rotational invariance of the signal subspaces corresponding to the underlying subarrays is then investigated based on the coprime coarray covariance matrix, and the incorporation of ESPRIT in the coarray domain makes it feasible to formulate the closed-form solution for DOA estimation. Theoretical analyses and simulation results verify the efficiency and the effectiveness of the proposed DOA estimation algorithm.

## 1. Introduction

Direction-of-arrival (DOA) estimation aims at retrieving the directional information of sources from the array received signals, and plays a fundamental role in a variety of practical application fields including radar, sonar, acoustics, radio astronomy, and wireless communications [1,2,3,4,5,6,7,8,9,10,11]. Restricted by the conditions revealed by the Nyquist sampling theorem, uniform linear array (ULA) becomes the most popular array geometry throughout the related research efforts in the past few decades, such that the Nyquist sampling rate can be fulfilled for resolving the DOAs in an unambiguous manner [12,13,14,15,16,17]. Nevertheless, the conventional algorithms using ULA fail to perform accurate DOA estimation when the number of sources exceeds the number of sensors, since the degrees-of-freedom (DOFs) are constrained by the number of sensors in the array. Moreover, the available sensors in the ULA also limit the array aperture that determines the estimation resolution. In order to cope with multiple sources while maintaining a high resolution, which are typical requirements for the ultra-dense cellular networks under the background of 5G communications [18], the massive sensors in the ULA as well as the associated radio frequency modules lead to a high computational complexity and hardware cost.

Recently, the coprime array has been proposed as the realization of coprime sampling in the spatial domain, and attracted tremendous interests in the field of array signal processing [19]. On one hand, as compared to the conventional sparse arrays such as the minimum redundancy arrays [20] and the minimum hole arrays [21], the systematic design of the coprime array has a more concise and flexible geometry for sparse array configuration. On the other hand, the coprimality enables increasing the achievable DOFs as well as the resolution without increasing the number of sensors. Therefore, these properties of coprime array provide a good balance between the estimation performance and the complexity concerns, triggering the research on coprime sensor array signal processing to explore the potential advantages beyond the Nyquist sampling rate, e.g., DOA estimation [22,23,24], adaptive beamforming [25,26,27,28], and spectrum estimation [29,30].

The MUltiple SIgnal Classification (MUSIC) technique [31] is one of the most important methods for coprime array DOA estimation, where an increased number of DOFs can be achieved via processing the equivalent coprime coarray signals [32]. However, the spectrum peak search process is a necessary step for retrieving the DOAs from the MUSIC spatial spectrum, leading to a high computational complexity if we set a small searching interval for high-resolution DOA estimation. The sparsity-based techniques are another kind of representative method for DOA estimation exploiting coprime array [33]. By incorporating the sparsity of the spatial sources, the equivalent signal of the derived coprime coarray is processed for DOA estimation with an increased number of DOFs via some criteria, such as the sparse signal reconstruction [34] and covariance matrix sparse reconstruction [35]. However, the predefined spatial sampling grids are a necessary condition for the optimization problem design in these algorithms, leading to an inherent DOA estimation bias, which is referred to as the basis mismatch, since the directions of the incident sources will not always be in accordance with these grids. Moreover, the computational complexity follows an exponential growth with the increment of the predefined spatial sampling grid density. To address the basis mismatch problem, several gridless algorithms have been proposed by reconstructing the covariance matrix of the derived coprime coarray via nuclear norm minimization [36] or trace minimization [37]. However, these MUSIC-like algorithms still estimate the DOAs from the MUSIC spatial spectrum, leading to the trade-off between the resolution performance and the computational complexity. Therefore, how to realize a computationally efficient DOA estimation while maintaining the advantages of the coprime array remains a challenging but promising problem.

In this paper, we propose a novel coprime array DOA estimation algorithm by processing the equivalent coprime coarray received signal with Estimation of Signal Parameters via Rotational Invariance Techniques (ESPRIT), where off-grid DOAs can be efficiently resolved without spectrum peak search. Unlike the ESPRIT-like algorithms carried out in [38,39], which perform ESPRIT based on the received signals of a coprime pair of sparse ULAs separately and obtain the unique solution from the phase ambiguities for DOA estimation according to the coprimality relationship revealed in [40], we consider here introducing ESPRIT to the coarray domain and seek the rotational invariance from the underlying coprime coarray, such that the DOF superiority of the coprime array can be maintained. In more detail, the coprime coarray with more virtual sensors than the practically deployed sensors is firstly derived as well as its corresponding second-order equivalent received signal. By selecting a pair of subarrays with the same ULA geometry from the coprime coarray, the shift invariance of the subarrays results in a rotational invariance relationship between their corresponding signal subspaces. Based on the derived coprime coarray covariance matrix, the rotational operator for the signal subspaces of the underlying subarrays is investigated, where the closed-form solution for DOA estimation is formulated via ESPRIT. With the incorporation of the ESPRIT in the coarray domain, neither the predefined spatial sampling grids nor the spectrum peak search process is required, indicating that the proposed coarray ESPRIT-based algorithm is capable of resolving off-grid DOAs with an increased number of DOFs. The computational complexity analyses are presented to evaluate the efficiency, and the simulations are conducted to demonstrate the effectiveness of the proposed DOA estimation algorithm.

The main contributions of this paper can be summarized as follows:

- We derive the coarray statistics of the coprime array, and introduce the idea of ESPRIT to the coarray domain for retrieving the DOAs with an increased number of DOFs.
- We extract a pair of shift invariant uniform linear subarrays from the coprime coarray, and investigate the rotational invariance of the corresponding coarray domain signal subspaces.
- We provide the closed-form solution for efficient DOA estimation, which enables performing off-grid DOA estimation without predefined spatial sampling grids or the spectrum peak search process.

The rest of this paper is organized as follows. Section 2 formulates the coprime array configuration and the received signal model. Section 3 details the proposed coarray ESPRIT-based DOA estimation algorithm, and Section 4 presents the simulation results for comparison. Finally, Section 5 draws the conclusions for this paper.

*Notations*: Throughout this paper, vectors and matrices are respectively represented by lower-case boldface and upper-case boldface characters. ${\left(\;\cdot \;\right)}^{*}$, ${\left(\;\cdot \;\right)}^{T}$, and ${\left(\;\cdot \;\right)}^{H}$ denote the conjugate, transpose, and conjugate transpose operator, respectively. E denotes the statistical expectation operator, vec(·) is the vectorization process, and ⊗ denotes the Kronecker product. ${\left(\;\cdot \;\right)}^{-1}$ and ${\left(\;\cdot \;\right)}^{†}$ denote the inverse and the pseudo-inverse, respectively. Finally, 0 and I respectively denote the zero vector and identity matrix with appropriate dimensions.

## 2. Coprime Array and Signal Model

**Figure 1:**
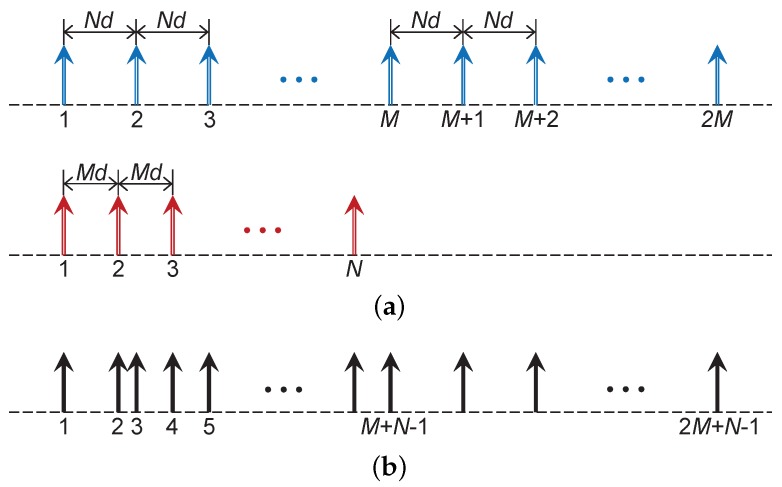
Illustration of the coprime array configuration. (**a**) the two uniform linear arrays for constructing the coprime array; (**b**) the non-uniform coprime array.

The coprime array consists of two ULAs as illustrated in Figure 1a, where *M* and *N* are coprime integers with M<N. The upper ULA has 2M sensors spaced Nd apart, i.e., $S_{1}=\left\{0,Nd,2Nd,\cdots ,\left(2M-1\right)Nd\right\}$, whereas the bottom ULA has *N* sensors spaced Md apart, i.e., $S_{2}=\left\{0,Md,2Md,\cdots ,\left(N-1\right)Md\right\}$. Here, *d* equals a half-wavelength. Collocating these two ULAs with the sensor at the zeroth position aligned, as shown in Figure 1b, the rest of the sensors do not overlap due to the coprimality. Therefore, the resulting non-uniform linear array $S=S_{1}\cup S_{2}$, which is referred to as the coprime array, consists of 2M+N-1 sensors in total.

Assuming there are *K* uncorrelated narrowband plane-wave signals from the directions $\theta ={\left[\theta_{1},\theta_{2},\cdots ,\theta_{K}\right]}^{T}$ impinging on the coprime array, the received signals at the *l*-th snapshot can be modeled as

(1) $$x\left(l\right)=\sum_{k=1}^{K}a\left(\theta_{k}\right)s_{k}\left(l\right)+n\left(l\right)=A\left(\theta \right)s\left(l\right)+n\left(l\right),$$

where $A\left(\theta \right)=\left[a\left(\theta_{1}\right),a\left(\theta_{2}\right),\cdots ,a\left(\theta_{K}\right)\right]\in C^{(2M+N-1)\times K}$ is the coprime array manifold matrix with the *k*-th column

(2) $$a\left(\theta_{k}\right)={\left[1,e^{-j\frac{2\pi }{\lambda }p_{2}\sin\left(\theta_{k}\right)},e^{-j\frac{2\pi }{\lambda }p_{3}\sin\left(\theta_{k}\right)},\cdots ,e^{-j\frac{2\pi }{\lambda }p_{2M+N-1}\sin\left(\theta_{k}\right)}\right]}^{T}$$

representing the manifold vector corresponding to $\theta_{k}$. Here, $j=\sqrt{-1}$ denotes the unit imaginary number, λ denotes the signal wavelength, and the set $S=\{p_{1},p_{2},p_{3},\cdots ,p_{2M+N-1}\}$ contains the locations of each sensor in the coprime array, whose first sensor is placed at the zeroth position, i.e., $p_{1}=0$. $s\left(l\right)={\left[s_{1}\left(l\right),s_{2}\left(l\right),\cdots ,s_{K}\left(l\right)\right]}^{T}$ contains the signal waveforms of each source, $n\left(l\right)∼\mathrm{CN}\left(0,\sigma_{n}^{2}I\right)$ is the complex-valued additive white Gaussian noise term, and $\sigma_{n}^{2}$ denotes the noise power.

The theoretical covariance matrix of the coprime array received signals x(l) is defined as

(3) $$R_{\mathrm{xx}}=E\left[x\left(l\right)x^{H}\left(l\right)\right]=\sum_{k=1}^{K}\sigma_{k}^{2}a\left(\theta_{k}\right)a^{H}\left(\theta_{k}\right)+\sigma_{n}^{2}I=A\left(\theta \right)\Sigma A^{H}\left(\theta \right)+\sigma_{n}^{2}I,$$

where $\Sigma =E\left[s\left(l\right)s^{H}\left(l\right)\right]=\mathrm{diag}\left(\left[\sigma_{1}^{2},\sigma_{2}^{2},\cdots ,\sigma_{K}^{2}\right]\right)$ is a K×K dimensional diagonal matrix formed with the power of the *K* sources $\sigma^{2}={\left[\sigma_{1}^{2},\sigma_{2}^{2},\cdots ,\sigma_{K}^{2}\right]}^{T}$ on its diagonal. Since the theoretical covariance matrix $R_{\mathrm{xx}}$ is unavailable in practice, it is usually approximated by the sample covariance matrix calculated from the *L* available snapshots as

(4) $${\hat{R}}_{\mathrm{xx}}=\frac{1}{L}\sum_{l=1}^{L}x\left(l\right)x^{H}\left(l\right).$$

Obviously, the sample covariance matrix ${\hat{R}}_{\mathrm{xx}}$ approaches its theoretical version $R_{\mathrm{xx}}$ when the number of snapshots tends to infinity.

## 3. The Proposed DOA Estimation Algorithm

In this section, we elaborate on the proposed coarray ESPRIT-based DOA estimation algorithm for coprime array, where off-grid DOA can be resolved without the predefined spatial sampling grids or the spectrum peak search process. The coprime array received signals are firstly transformed to the equivalent second-order received signal of an augmented coprime coarray, such that the number of DOFs can be effectively increased. Since the ESPRIT requires a shift invariant array geometry to perform, a pair of uniform linear subarrays is extracted from the derived coprime coarray, where the shift invariance is investigated based on the signal statistics in the coarray domain. Based on the rotational invariance of the subspaces corresponding to the subarray pair, the closed-form solution is formulated for efficiently estimating the DOAs of each source.

### 3.1. Coprime Coarray Statistics Derivation

The coarray domain equivalent received signal can be derived by vertically stacking each column of the sample covariance matrix ${\hat{R}}_{\mathrm{xx}}$ as

(5) $$y=\mathrm{vec}\left({\hat{R}}_{\mathrm{xx}}\right)=C\sigma^{2}+\sigma_{n}^{2}i,$$

where i=vec(I), and $C=\left[a^{*}\left(\theta_{1}\right)⊗a\left(\theta_{1}\right),a^{*}\left(\theta_{2}\right)⊗a\left(\theta_{2}\right),\cdots ,a^{*}\left(\theta_{K}\right)⊗a\left(\theta_{K}\right)\right]\in C^{{\left(2M+N-1\right)}^{2}\times K}$ is the manifold matrix of an augmented virtual array, whose virtual sensor locations are given by

(6) $$S_{V}=\left\{p_{ı}-p_{ȷ},ı,ȷ=1,2,\cdots ,2M+N-1\right\}.$$

Since the elements $a^{*}\left(\theta_{k}\right)⊗a\left(\theta_{k}\right)$ in C are in the form of $\left\{e^{-j\frac{2\pi }{\lambda }\left(p_{ı}-p_{ȷ}\right)\sin\left(\theta_{k}\right)},ı,ȷ=1,2,\cdots ,2M+N-1\right\}$, y can be viewed as the received signal of the augmented virtual array $S_{V}$, whose unique elements yield the coprime coarray as

(7) $$S_{C}=\left\{\pm \left(Mn-Nm\right)d,0\leq m\leq 2M-1,0\leq n\leq N-1\right\}.$$

Although the coprime coarray is non-uniform, it has been revealed in [23] that the coprime array configuration we deployed in Figure 1b is a special case of the generalized coprime array with compressed inter-element spacing configuration, and it has been proved in [23] that there are 2MN+2M-1 continuous virtual sensors in $S_{C}$ locating from (-MN-M+1)d to (MN+M-1)d, which is referred to as the continuous part of the coprime array. While the ESPRIT requires a shift invariant array geometry to explore the rotational invariance, we focus on the continuous part of the coprime coarray for the proposed algorithm, such that the Nyquist sampling theorem can be fulfilled and the ESPRIT can be introduced to the coarray domain to achieve an increased number of DOFs. Accordingly, the equivalent received signal of the continuous coprime coarray can be obtained by selecting the elements corresponding to these virtual sensors from y, modeling as

(8) $$\tilde{y}=\tilde{C}\sigma^{2}+\sigma_{n}^{2}\tilde{i},$$

where $\tilde{C}\in C^{(2MN+2M-1)\times K}$ denotes the manifold matrix of the continuous coprime coarray, and $\tilde{i}$ contains the corresponding elements in i.

On the other hand, although $\tilde{y}$ has a similar form as the received signal model with a ULA geometry, it actually belongs to the second-order statistics containing the power of each source $\sigma^{2}$ rather than the signal waveforms s(l) as in Equation (1). Therefore, the equivalent received signal $\tilde{y}$ behaves in a single snapshot manner in the coarray domain, resulting in the rank deficient problem for the corresponding sample covariance matrix, and the multiple sources cannot be effectively estimated with such a rank-one coarray domain covariance matrix. To address this problem, the spatial smoothing techniques can be applied for rank enhancement [32,35]. In particular, the continuous part of the coprime coarray is divided into MN+M overlapping subarrays, and the *ℓ*-th subarray consists of MN+M virtual sensors locating from (-ℓ+1)d to (-ℓ+MN+M)d, where the corresponding (MN+M)-dimensional second-order received signals of the MN+M subarrays can be viewed as the MN+M snapshots in the coarray domain. Collecting these subarray signals and calculating the correlations yield the spatially smoothed covariance matrix as

(9) $${\tilde{R}}_{\mathrm{ss}}=\frac{1}{MN+M}\sum_{\ell =1}^{MN+M}{\tilde{y}}_{\ell }{\tilde{y}}_{\ell }^{H},$$

where ${\tilde{y}}_{\ell }\in C^{MN+M}$ contains the (MN+M+1-ℓ)-th to the (2MN+2M-ℓ)-th elements in $\tilde{y}$. Since the (MN+M)×(MN+M) dimensional spatially smoothed covariance matrix ${\tilde{R}}_{\mathrm{ss}}$ is a summation of the correlations from the MN+M coarray domain snapshots, it is now a full rank matrix, which is capable of resolving up to MN+M-1 sources. Accordingly, the relationship between the fourth-order spatially smoothed covariance matrix ${\tilde{R}}_{\mathrm{ss}}$ and the coprime coarray covariance matrix ${\tilde{R}}_{\mathrm{yy}}$ is [32]

(10) $${\tilde{R}}_{\mathrm{ss}}=\frac{1}{MN+M}{\tilde{R}}_{\mathrm{yy}}^{2}.$$

Here, the theoretical coprime coarray covariance matrix can be represented as

(11) $$R_{\mathrm{yy}}={\tilde{C}}_{1}\Sigma {\tilde{C}}_{1}^{H}+\sigma_{n}^{2}I,$$

where ${\tilde{C}}_{1}\in C^{(MN+M)\times K}$ denotes the manifold matrix of the virtual ULA consisting of MN+M virtual sensors ranging from 0 to (MN+M-1)d, indicating that the achievable DOFs for DOA estimation can reach up to MN+M-1 by only using 2M+N-1 sensors. However, the theoretical version of ${\tilde{R}}_{\mathrm{yy}}$ in Equation (11) is practically unavailable, and the square root operation based on the spatially smoothed covariance matrix ${\tilde{R}}_{\mathrm{ss}}$ in Equation (10) is also indefinite. Encouragingly, a definite representation for the coprime coarray covariance matrix ${\tilde{R}}_{\mathrm{yy}}$ has been proved in [41] by rearranging the elements of $\tilde{y}$ in a Toeplitz matrix structure as

(12) $${\tilde{R}}_{\mathrm{yy}}=\left[\begin{array}{cccc} {\langle \tilde{y}\rangle }_{0} & {\langle \tilde{y}\rangle }_{-1} & \cdots & {\langle \tilde{y}\rangle }_{-MN-M+1}\\ {\langle \tilde{y}\rangle }_{1} & {\langle \tilde{y}\rangle }_{0} & \cdots & {\langle \tilde{y}\rangle }_{-MN-M+2}\\ \;\;\vdots & \;\;\vdots & \ddots & \;\;\vdots \\ {\langle \tilde{y}\rangle }_{MN+M-1} & {\langle \tilde{y}\rangle }_{MN+M-2} & \cdots & {\langle \tilde{y}\rangle }_{0} \end{array}\right],$$

where ${\langle \;\cdot \;\rangle }_{\ell }$ denotes the received signal corresponding to the virtual sensor located at ℓd. While operating the (MN+M)×(MN+M) dimensional full rank matrix ${\tilde{R}}_{\mathrm{yy}}$ enables achievable DOFs up to MN+M-1 by only using 2M+N-1 sensors, we propose introducing ESPRIT to the coarray domain and investigating the rotational invariance based on the coprime coarray covariance matrix ${\tilde{R}}_{\mathrm{yy}}$.

### 3.2. ESPRIT in Coarray Domain for DOA Estimation

**Figure 2:**
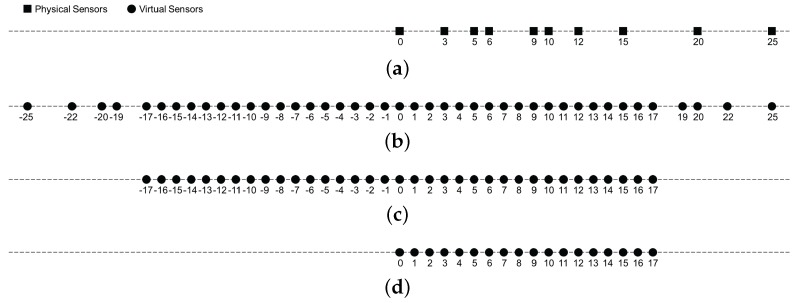
Illustration of each array configuration with an example of M=3 and N=5, the unit spacing is *d*. (**a**) coprime array; (**b**) coprime coarray; (**c**) continuous part of coprime coarray; (**d**) the equivalent virtual ULA corresponding to the coprime coarray covariance matrix.

To employ the ESPRIT in the coarray domain, a pair of translationally separated coprime coarray subarrays with the same geometry is required, such that the corresponding coarray statistic characteristics imposed by the shift invariance can be investigated. While the mathematically derived coprime coarray is practically nonexistent, the available statistics in the coarray domain are the second-order received signal y and the coprime coarray covariance matrix ${\tilde{R}}_{\mathrm{yy}}$. In view of the continuous part of the coprime coarray, which is symmetrical to the zeroth position, the second-order received signal in $\tilde{y}$ corresponding to the virtual sensors locate at ℓd and -ℓd are mutually conjugate based on the elements in C. Therefore, the coprime coarray covariance matrix ${\tilde{R}}_{\mathrm{yy}}$ can be represented in a more compact form as

(13) $${\tilde{R}}_{\mathrm{yy}}=T\left(u\right)=\left[\begin{array}{cccc} {\langle \tilde{y}\rangle }_{0} & {\langle \tilde{y}\rangle }_{1}^{*} & \cdots & {\langle \tilde{y}\rangle }_{MN+M-1}^{*}\\ {\langle \tilde{y}\rangle }_{1} & {\langle \tilde{y}\rangle }_{0} & \cdots & {\langle \tilde{y}\rangle }_{MN+M-2}^{*}\\ \;\;\vdots & \;\;\vdots & \ddots & \;\;\vdots \\ {\langle \tilde{y}\rangle }_{MN+M-1} & {\langle \tilde{y}\rangle }_{MN+M-2} & \cdots & {\langle \tilde{y}\rangle }_{0} \end{array}\right],$$

where T(u) denotes the Hermitian Toeplitz matrix with vector u as its first column, and $u={\left[{\langle \tilde{y}\rangle }_{0},{\langle \tilde{y}\rangle }_{1},\cdots ,{\langle \tilde{y}\rangle }_{MN+M-1}\right]}^{T}$. The Hermitian Toeplitz form of ${\tilde{R}}_{\mathrm{yy}}$ in Equation (13) confirms the fact that the coprime coarray covariance matrix is relevant to the second-order statistics corresponding to the virtual ULA ranging from 0 to (MN+M-1)d. To have an intuitive impression, we illustrate the geometry of the coprime array, coprime coarray, and continuous coprime coarray, the equivalent virtual ULA corresponding to the coprime coarray covariance matrix ${\tilde{R}}_{\mathrm{yy}}$ in Figure 2 with an example of M=3 and N=5.

**Figure 3:**
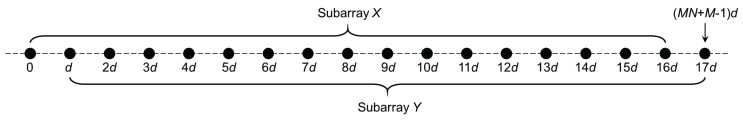
The pair of subarrays $S_{X}$ and $S_{Y}$ extracted from the coprime coarray, M=3 and N=5.

Based on the observations mentioned above, a pair of uniform linear subarrays consisting of MN+M-1 virtual sensors are extracted from the coprime coarray with the locations respectively at $S_{X}=\left\{0,d,2d,\cdots ,\left(MN+M-2\right)d\right\}$ and $S_{Y}=\left\{d,2d,3d,\cdots ,\left(MN+M-1\right)d\right\}$, which are illustrated in Figure 3. Obviously, both of the subarrays belong to the ULA, where a known translationally separated displacement space of *d* is existed for the corresponding virtual sensors in each subarray.

Similar to Equation (1), the received signals of the pair of coprime coarray subarrays can be theoretically modeled as

(14) $$z_{X}\left(l\right)={\tilde{C}}_{1,X}s\left(l\right)+n_{X}\left(l\right),$$

and

(15) $$z_{Y}\left(l\right)={\tilde{C}}_{1,Y}s\left(l\right)+n_{Y}\left(l\right),$$

where ${\tilde{C}}_{1,X}$ and ${\tilde{C}}_{1,Y}$ are (MN+M-1)×K dimensional manifold matrices, respectively, corresponding to $S_{X}$ and $S_{Y}$, and $n_{X}\left(l\right)$ and $n_{Y}\left(l\right)$ are the noise terms. Here, the manifold matrices ${\tilde{C}}_{1,X}$ and ${\tilde{C}}_{1,Y}$ can be respectively obtained by removing the last row and the first row of ${\tilde{C}}_{1}$ in Equation (11), namely,

(16) $${\tilde{C}}_{1}=\left[\begin{array}{c} \mathrm{diag}(I)\\ \;\;\;{\tilde{C}}_{1,X} \end{array}\right]=\left[\begin{array}{c} \;\;{\tilde{C}}_{1,Y}\\ e^{-j\frac{2\pi }{\lambda }\left(MN+M-1\right)d\sin\theta^{T}} \end{array}\right].$$

While the subarray pair $S_{X}$ and $S_{Y}$ share an identical ULA geometry except the inherent displacement spacing of *d* between the doublet virtual sensors, the received signals $z_{Y}\left(l\right)$ in Equation (15) can be equivalently represented with respect to the manifold matrix of $S_{X}$ as

(17) $$z_{Y}\left(l\right)={\tilde{C}}_{1,X}\Phi s\left(l\right)+n_{Y}\left(l\right),$$

where

(18) $$\Phi =\mathrm{diag}\left\{e^{-j\frac{2\pi }{\lambda }d\sin\left(\theta_{1}\right)},e^{-j\frac{2\pi }{\lambda }d\sin\left(\theta_{2}\right)},\cdots ,e^{-j\frac{2\pi }{\lambda }d\sin\left(\theta_{K}\right)}\right\}$$

is a K×K dimensional unitary matrix that relates the received signals from the two subarrays $S_{X}$ and $S_{Y}$, indicating the shift invariance of the two subarrays. Accordingly, the theoretical covariance matrices corresponding to the pair of coprime coarray subarrays $z_{X}\left(l\right)$ and $z_{Y}\left(l\right)$ are

(19) $$R_{z_{X}z_{X}}={\tilde{C}}_{1,X}\Sigma {\tilde{C}}_{1,X}^{H}+\sigma_{n}^{2}I,$$

and

(20) $$R_{z_{Y}z_{Y}}={\tilde{C}}_{1,Y}\Sigma {\tilde{C}}_{1,Y}^{H}+\sigma_{n}^{2}I={\tilde{C}}_{1,X}\Phi \Sigma \Phi^{H}{\tilde{C}}_{1,X}^{H}+\sigma_{n}^{2}I,$$

respectively.

The shift invariance of the coprime coarray subarray pair results in the rotational invariance of the underlying signal subspaces in $R_{z_{X}z_{X}}$ and $R_{z_{Y}z_{Y}}$, and the basic idea of ESPRIT can thus be readily adopted in the coarray domain. Nevertheless, the theoretical covariance matrices $R_{z_{X}z_{X}}$ and $R_{z_{Y}z_{Y}}$ are unavailable due to the finite snapshots in the coarray domain. Moreover, the first-order coarray received signals $z_{X}\left(l\right)$ and $z_{Y}\left(l\right)$ containing the signal waveforms are also unavailable, since the virtual sensors exist in a mathematical sense and never receive the signal waveforms in practice. In this regard, we consider investigating the rotational invariance of the subarray pair subspaces based on the derived coprime coarray covariance matrix ${\tilde{R}}_{yy}$, and the eigen-decomposition of ${\tilde{R}}_{yy}$ can be represented as

(21) $${\tilde{R}}_{yy}=\Omega_{S}\Lambda_{S}\Omega_{S}^{H}+\Omega_{N}\Lambda_{N}\Omega_{N}^{H},$$

where $\Omega_{S}\in C^{(MN+M)\times K}$ denotes the signal subspace collecting from the eigenvectors of the *K* largest eigenvalues contained in the diagonal of $\Lambda_{S}$. Similarly, the eigenvectors corresponding to the remaining MN+M-K eigenvalues of ${\tilde{R}}_{yy}$ in the diagonal of $\Lambda_{N}$ form the noise subspace $\Omega_{N}$. While the signal subspace of the coprime coarray covariance matrix ${\tilde{R}}_{yy}$ is mapped to the virtual ULA ranging from 0 to (MN+M)d that contains the pair of coprime coarray subarrays, the signal subspaces corresponding to $S_{X}$ and $S_{Y}$ can be generated by removing the first row and the last row in $\Omega_{S}$, respectively. In particular, the (MN+M-1)×K dimensional signal spaces $\Omega_{S,X}$ and $\Omega_{S,Y}$ can be represented as

(22) $$\Omega_{S}=\left[\begin{array}{c} \Omega_{S}\left(1,:\right)\\ \;\;\;\Omega_{S,X} \end{array}\right]=\left[\begin{array}{c} \;\;\;\;\Omega_{S,Y}\\ \Omega_{S}\left((MN+M),:\right) \end{array}\right].$$

Since the column space of $\Omega_{S,X}$ shares the same subspace spanned by the columns of the coarray manifold matrix ${\tilde{C}}_{1,X}$, there exists a unique K×K dimensional nonsingular matrix V satisfying

(23) $$\Omega_{S,X}={\tilde{C}}_{1,X}V.$$

Combining the relationship revealed in Equation (17), which is imposed by the shift invariance, we have

(24) $$\Omega_{S,Y}={\tilde{C}}_{1,Y}V={\tilde{C}}_{1,X}\Phi V.$$

Since the column spaces spanned by $\Omega_{S,X}$ and $\Omega_{S,Y}$ are identical, the rank of the matrix $\Omega_{S,\mathrm{XY}}=\left[\Omega_{S,X}\;\;\;\Omega_{S,Y}\right]$ remains *K*. Hence, there exists a 2K×K dimensional full rank matrix $Q=\left[Q_{X};Q_{Y}\right]$, which is orthogonal to the matrix $\Omega_{S,\mathrm{XY}}$, namely,

(25) $$\Omega_{S,\mathrm{XY}}Q=\left[\Omega_{S,X}\;\;\;\Omega_{S,Y}\right]\left[\begin{array}{c} Q_{X}\\ Q_{Y} \end{array}\right]=\Omega_{S,X}Q_{X}+\Omega_{S,Y}Q_{Y}=0.$$

Substituting the relationship between the signal space and the manifold matrix established in Equation (23), Equation (25) can be rewritten as

(26) $$-{\tilde{C}}_{1,X}VQ_{X}Q_{Y}^{-1}={\tilde{C}}_{1,X}\Phi V.$$

Defining $\Psi =-Q_{X}Q_{Y}^{-1}$, we have the following relationship indicating the rotational invariance between the signal subspaces of the pair of coprime coarray subarrays $\Omega_{S,X}$ and $\Omega_{S,Y}$ as

(27) $$\Omega_{S,Y}=\Omega_{S,X}\Psi ,$$

where the rotational operator between the signal spaces $\Omega_{S,X}$ and $\Omega_{S,Y}$ can be calculated as

(28) $$\Psi =\Omega_{S,X}^{†}\Omega_{S,Y},$$

and Equation (26) can be transformed to

(29) $${\tilde{C}}_{1,X}V\Psi ={\tilde{C}}_{1,X}\Phi V.$$

While the matrix V is invertible and ${\tilde{C}}_{1,X}$ is a full rank matrix, we have

(30) $$\Phi =V\Psi V^{-1}.$$

Hence, Φ and Ψ are similar matrices, which share the same eigenvalue. Since the DOAs of the incident sources are contained in Φ according to Equation (18), the closed-form DOA estimation for the *k*-th source can be readily formulated as

(31) $${\hat{\theta }}_{k}=\arcsin\left(-\frac{1}{\pi }ℑ\left(\ln\psi_{k}\right)\right),\;k=1,2,\cdots ,K,$$

where $\psi_{k}$ is the *k*-th eigenvalue of Ψ, and ℑ(·) denotes the imaginary part of a complex number.

### 3.3. Computational Complexity Analyses and Remarks

The computational complexity of the proposed DOA estimation algorithm is $O({\left(MN+M-1\right)}^{3})$, which is dominated by the eigenvalue decomposition process of the coprime coarray covariance matrix ${\tilde{R}}_{yy}$. By contrast, the sparse signal reconstruction algorithm [34] has a computational complexity of $O({\left(2M+N-1\right)}^{2}G)$, where G≫2M+N-1 is the number of predefined spatial sampling grids for the sparse signal reconstruction optimization problem. Obviously, the computational complexity grows exponentially with the spatial sampling grids being denser, and the trade-off between the resolution capability and the computational complexity is encountered. Although the spatial smoothing MUSIC algorithm [32] do not require the predefined spatial sampling grids as a necessary condition, its computational complexity $O({\left(MN+M\right)}^{2}S)$ is dominated by the spectrum peak search process, and the number of hypothetical directions S≫MN+M is usually much larger than the number of sources *K* to ensure the estimation resolution and accuracy. Therefore, the proposed coarray ESPRIT-based algorithm has a superior performance in terms of computational complexity, and enables efficiently resolving off-grid DOAs.

The steps of the proposed coarray ESPRIT-based DOA estimation algorithm are listed in Table 1, whose main advantages can be summarized as follows: first, we introduce ESPRIT to the coarray domain, and investigate the rotational invariance based on a pair of shift invariant uniform linear coprime coarray subarrays, such that the difficulties caused by the non-uniformity of the coprime array can be overcome, and the available DOF is effectively increased in the meantime. Second, considering the fact that the coarray domain received signals $z_{X}\left(l\right)$ and $z_{Y}\left(l\right)$ are practically unavailable due to the mathematically derived virtual sensors in the coprime coarray, the signal subspaces of the pair of coprime coarray subarrays $\Omega_{S,X}$ and $\Omega_{S,Y}$ are formulated based on the coprime coarray covariance matrix ${\tilde{R}}_{yy}$, enabling the investigation of the rotational invariance imposed by the shift invariant subarrays in the coarray domain. Last but not least, neither the predefined spatial sampling grids nor the spectrum peak search process is required for the proposed algorithm, indicating that the proposed algorithm enables estimating off-grid DOAs in an efficient manner.

## 4. Simulation Results

In our simulations, the pair of coprime integers is selected to be M=3 and N=5, indicating that 2M+N-1=10 sensors are utilized to deploy the coprime array with the locations at S={0,3d,5d,6d,9d,10d,12d,15d,20d,25d} (except one simulation in the last example, where the number of sensors in the coprime array is varied). The performance of the proposed coarray ESPRIT-based DOA estimation algorithm is compared to several DOA estimation algorithms exploiting coprime array, including the sparse signal reconstruction (SSR) algorithm [34], the spatial smoothing MUSIC algorithm (SS-MUSIC) [32], and the coprime virtual array interpolation-based algorithm [37]. The predefined spatial sampling grids for the SSR algorithm are from $\left[-90^{\circ },90^{\circ }\right]$ with the sampling interval being $0.1^{\circ }$, whereas the spectrum peak search process for the SS-MUSIC algorithm and the coprime virtual array interpolation-based algorithm is also within $\left[-90^{\circ },90^{\circ }\right]$ with the searching interval of $0.1^{\circ }$. The regularization parameter for the SSR algorithm and the coprime virtual array interpolation-based algorithm is empirically chosen to be 0.25, which is recommended in the respective literatures.

**Figure 4:**
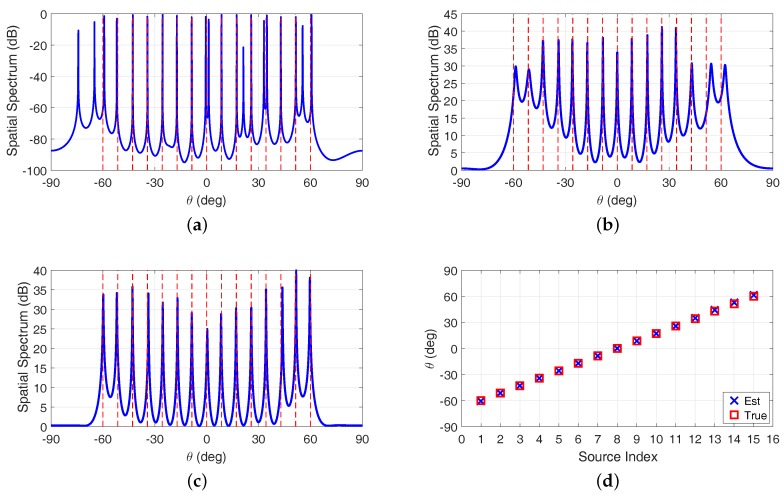
Direction-of-arrival (DOA) estimation performance of each algorithm using coprime array when there are more sources than sensors. (**a**) sparse signal reconstruction (SSR) algorithm; (**b**) spatial smoothing multiple signal classification (SS-MUSIC) algorithm; (**c**) coprime virtual array interpolation-based algorithm; (**d**) proposed algorithm.

In the first example, we compare the DOA estimation performance of each algorithm using coprime array in Figure 4 by assuming there are more sources than sensors, where the directions of 15 sources are uniformly distributed within $\left[-60^{\circ },60^{\circ }\right]$. The spatial spectra of the SSR algorithm, the SS-MUSIC algorithm and the coprime virtual array interpolation-based algorithm are depicted in Figure 4a–c, respectively. While the proposed coarray ESPRIT-based algorithm does not estimate the sources power or calculate the spatial spectrum, we present the estimated DOAs of the proposed algorithm on the *y*-axis of Figure 4d with respect to the source index instead. The Signal-to-Noise Ratio (SNR) is 0 dB with the number of snapshots L=500. The true DOAs are illustrated by the vertical red dashed lines in Figure 4a–c, and the red rectangular markers in Figure 4d, respectively.

It is clear that all of the algorithms we considered are capable of identifying the 15 sources with only 10 sensors, where the DOF superiority offered by the coprime array is demonstrated. The additional peaks appeared in the spatial spectrum of the SSR algorithm are caused by the undetermined regularization parameter, which is utilized to balance the sparsity and the reconstruction accuracy in the optimization problem for signal reconstruction. These irregular spurious peaks, especially those that closely approach the peaks corresponding to the true DOAs, lead to the difficulties in determining DOA estimations. The coprime virtual array interpolation-based algorithm has a better spatial spectrum characteristic than the SS-MUSIC algorithm since the array interpolation process makes full use of the information contained in the non-uniform coprime coarray $S_{C}$. Due to the different algorithm design principle, the proposed algorithm formulates the closed-form DOA estimations by investigating the rotational invariance in the coarray domain, rather than searching the peaks in the calculated spatial spectrum for DOA estimation as those in the MUSIC-like algorithms. Therefore, the high complexity spectrum peak search process can be avoided, and off-grid DOA estimation can thus be realized via the incorporation of ESPRIT in the coarray domain. Although the proposed algorithm cannot simultaneously estimate the sources power, we would emphasize that the MUSIC spatial spectrum is a typical pseudo-spectrum, indicating that the spectrum responses in the MUSIC spatial spectrum cannot reflect the actual sources power. The performance of the proposed algorithm shown in Figure 4d indicate that exploiting ESPRIT in the coarray domain can increase the DOFs for DOA estimation, demonstrating the successful application of the ESPRIT in the non-uniform coprime array. In addition, we would emphasize that the conventional DOA estimation algorithms using ULA cannot resolve all of the sources in this scenario, since the available DOFs of the ULA-based algorithms are fundamentally limited by the number of sensors in the array.

In the second example, we compare the Root Mean Square Error (RMSE) of the estimated DOAs for each algorithm, where the RMSE criterion is defined as

(32) $$\mathrm{RMSE}=\sqrt{\frac{1}{QK}\sum_{q=1}^{Q}\sum_{k=1}^{K}{\left({\hat{\theta }}_{k,\;q}-\theta_{k}\right)}^{2}}.$$

**Figure 5:**
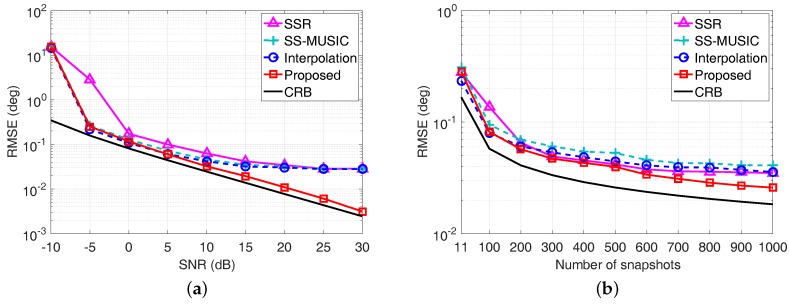
Root mean square error (RMSE) performance comparison with a single source, whose direction is randomly selected from $N\left(0^{\circ },1^{\circ }\right)$ in each Monte Carlo trial. (**a**) RMSE versus SNR with L=50; (**b**) RMSE versus the number of snapshots with SNR = 0 dB.

Here, ${\hat{\theta }}_{k,\;q}$ denotes the estimated DOA of the *k*-th source in the *q*-th Monte Carlo trial, and the RMSE is averaged from Q=500 Monte Carlo trials for each scenario. The RMSE of each algorithm is shown in Figure 5, where the direction of the incident source is randomly chosen from a standard normal distribution $N\left(0^{\circ },1^{\circ }\right)$. The direction of the random source varies from trial to trial, but remains fixed from snapshot to snapshot. Meanwhile, the Cramér–Rao Bound (CRB) is also plotted for reference.

We can observe from Figure 5a that the RMSE curve of the SSR algorithm becomes relatively flat in high SNRs, since the basis mismatch caused by the predefined spatial sampling grids limits the estimation accuracy. Similarly, the fixed searching interval for the MUSIC spectrum peak search process of both SS-MUSIC algorithm and the coprime virtual array interpolation-based algorithm limits the estimation accuracy, leading to the RMSE curve becoming flat when SNR is larger than 10 dB. By contrast, the RMSE performance of the proposed coarray ESPRIT-based algorithm outperforms the other algorithms and has a similar trend as the CRB when the SNR becomes large, since neither the predefined spatial sampling grids nor spectrum peak search process is required for the proposed algorithm. Meanwhile, the RMSE performance versus the number of snapshots depicted in Figure 5b also demonstrates the superiority of the proposed algorithm over the other algorithms, especially when the number of snapshots is larger than 500.

**Figure 6:**
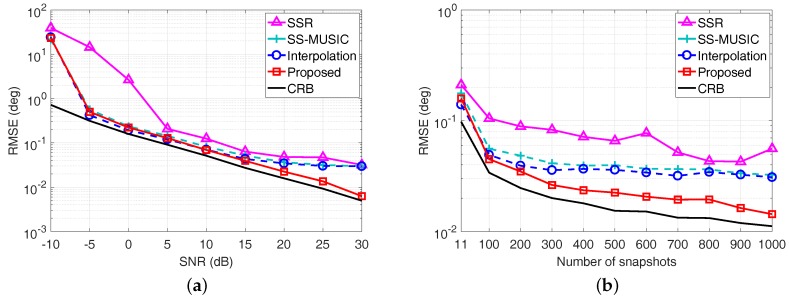
RMSE performance comparison with a single source, whose direction is randomly selected from $\left[-80^{\circ },80^{\circ }\right]$ in each Monte Carlo trial. (**a**) RMSE versus SNR with L=50; (**b**) RMSE versus the number of snapshots with SNR = 10 dB.

Meanwhile, we also consider a more generalized case, where the direction of the incident source is randomly selected from the uniform distribution on the interval $\left[-80^{\circ },80^{\circ }\right]$ in each Monte Carlo trial. The RMSE performance comparison of each algorithm versus the SNR and the number of snapshots are shown in Figure 6a, b, respectively. Similarity, the proposed algorithm still outperforms the compared algorithms especially in high SNRs. Therefore, the estimation accuracy superiority of the proposed coarray ESPRIT-based algorithm for resolving off-grid DOAs is verified.

**Figure 7:**
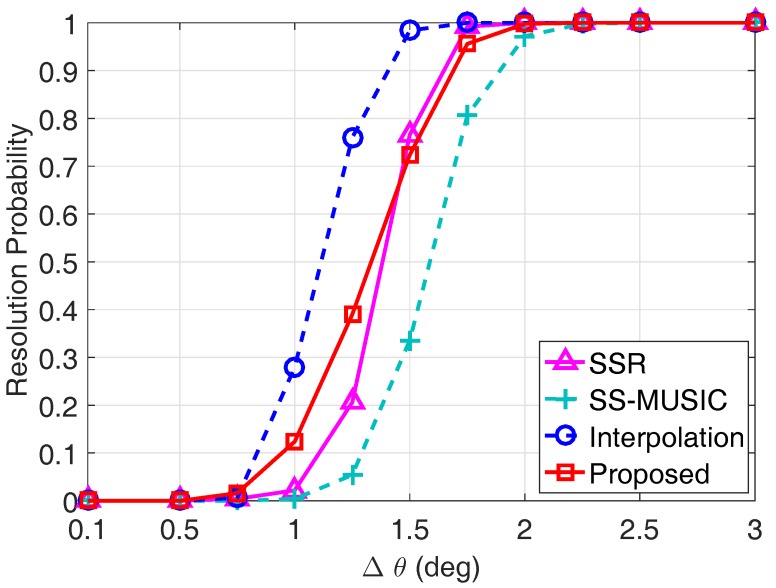
Resolution probability comparison of each algorithm with SNR = 0 dB and L=500.

In the third example, we compare the resolution performance of each algorithm in Figure 7 by assuming there are two closely spaced sources. The direction of the first source $\theta_{1}$ is randomly selected from the standard normal distribution $N\left(0^{\circ },1^{\circ }\right)$, which varies from trial to trial but is fixed from snapshot to snapshot, and the direction of the second source has an inherent angular spacing of Δθ with $\theta_{1}$, namely, $\theta_{2}=\theta_{1}+\Delta \theta$. The algorithm is identified to perform a successful DOA estimation if the absolute value of the bias for both estimated DOAs is smaller than Δθ/2 as compared to their respective true DOAs, namely, $|{\hat{\theta }}_{1}-\theta_{1}|<\Delta \theta /2$ and $|{\hat{\theta }}_{2}-\theta_{2}|<\Delta \theta /2$. The resolution probability is calculated from the percentage of the success trials among Q=500 Monte Carlo trials. The SNR is set to be 0 dB, and the number of snapshots is L=500.

It can be observed from Figure 7 that the proposed algorithm has a larger resolution probability than the SS-MUSIC algorithm, indicating that performing ESPRIT in the coarray domain can achieve a better resolution performance than the coarray MUSIC technique. Since the coprime virtual array interpolation-based algorithm utilizes all of the information contained in the non-uniform coprime coarray $S_{C}$, it achieves a better resolution probability than the proposed algorithm, which only utilizes the continuous part of the coprime coarray. Nevertheless, there exists a trade-off between the resolution probability and the computational complexity for the coprime virtual array interpolation-based algorithm, since the array interpolation-based optimization problem increases the computational complexity significantly. To have an intuitive understanding on the computational complexity, we list the computation time of each algorithm on an Intel Core i7-7600U, 16 GB RAM laptop in Table 2. Obviously, the computational time for the proposed algorithm is much less than the other algorithms. In particular, the computation time of the proposed algorithm only occupies 0.43% of the consumed time of the coprime virtual array interpolation-based algorithm, indicating the superiority of the proposed algorithm in terms of computational efficiency. Although the SSR algorithm also utilizes all of the information contained in the non-uniform coprime coarray $S_{C}$ for DOA estimation, its resolution probability is inferior to the proposed algorithm when Δθ is smaller than $1.5^{\circ }$. In addition, the SSR algorithm takes the maximum computation time among the simulated algorithms according to the results listed in Table 2. Therefore, the proposed coarray ESPRIT-based algorithm has a good balance between the resolution performance and the computational efficiency.

**Figure 8:**
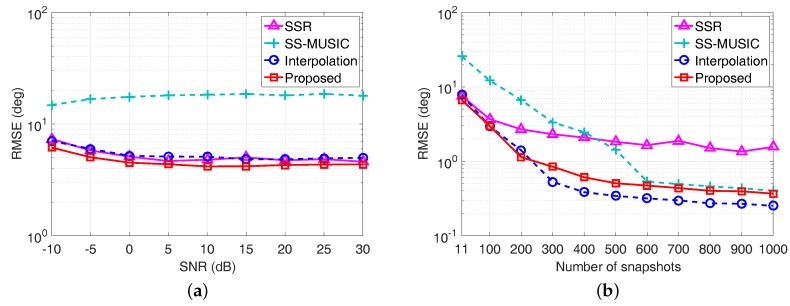
RMSE performance comparison when there are more sources than sensors. (**a**) RMSE versus SNR with L=50; (**b**) RMSE versus the number of snapshots with SNR = 0 dB.

In the fourth example, we compare the RMSE performance of each algorithm in Figure 8 when the number of sources exceeds the sensors, namely, there are 15 sources from the directions uniformly distributed in $\left[-60^{\circ },60^{\circ }\right]$. The SNR is fixed at 0 dB when we vary the number of snapshots, whereas the number of snapshots equals to L=50 when the SNR varies. For each scenario, 500 Monte Carlo trials are performed.

It is clear from Figure 8a that the RMSE of the proposed algorithm has the best performance when the available snapshots is relatively limited. Although the coprime virtual array interpolation-based algorithm outperforms the proposed algorithm when the number of snapshots is larger than 200 as shown in Figure 8b, the computational complexity brought by the array interpolation-based optimization problem as well as the matrix operation on the corresponding dimensionally extended covariance matrix result in a heavy computation burden. In particular, the computation time for 500 Monte Carlo trials of the SSR algorithm, the SS-MUSIC algorithm, the coprime virtual array interpolation-based algorithm, and the proposed algorithm on an Intel Core i7-7600U, 16 GB RAM laptop in this example is 1526.43 s, 9.71 s, 317.98 s, and 1.38 s, respectively. Therefore, exploiting ESPRIT in the coarray domain is capable of effectively resolving off-grid DOAs with an increased number of DOFs in an efficient manner.

**Figure 9:**
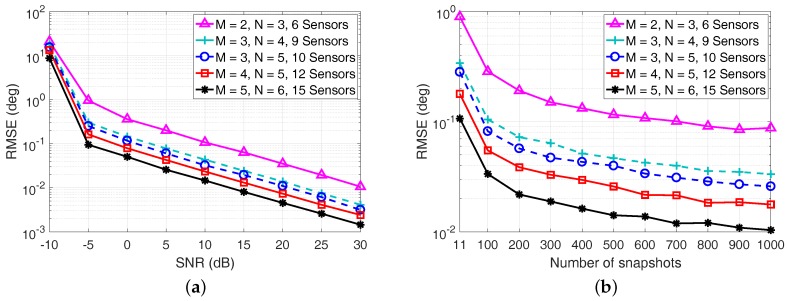
RMSE performance of the proposed algorithm with different numbers of sensors in the coprime array. (**a**) RMSE versus the SNR with L=50; (**b**) RMSE versus the number of snapshots with SNR = 0 dB.

In the last example, we depict the RMSE performance of the proposed algorithm in Figure 9 by differing the number of sensors in the coprime array. Five coprime array configurations are considered by selecting the coprime integer pair as (M=2,N=3), (M=3,N=4), (M=3,N=5), (M=4,N=5), (M=5,N=6), respectively. The other parameters are the same as those in Figure 5.

It is clear from Figure 9 that the RMSE of the proposed algorithm is getting smaller with the increase of the number of sensors in the coprime array. This is because a larger array aperture can be obtained for the coprime array when more sensors are available. In addition, more continuous virtual sensors in the coprime coarray can be utilized to perform ESPRIT for the proposed algorithm. Moreover, with the increase of the SNR and the number of snapshots, the RMSE of the proposed algorithm kept decreasing regardless of how many sensors we utilized, indicating the effectiveness of the proposed DOA estimation algorithm for different coprime array configurations.

## 5. Conclusions

We have proposed a novel coarray ESPRIT-based DOA estimation algorithm for coprime array, where off-grid DOAs can be efficiently resolved with increased number of DOFs. The coprime coarray is firstly derived as well as its corresponding statistics, where more virtual sensors can be utilized by processing the equivalent second-order received signal. By extracting a pair of shift invariant uniform linear subarrays from the coprime coarray, the rotational invariance of their signal subspaces is investigated in the coarray domain, and the closed-form solution for DOA estimation is formulated based on the idea of ESPRIT. Neither the predefined spatial sampling grids nor the spectrum peak search process is required for the proposed algorithm, ensuring a good balance between the resolution performance and the computational complexity. Theoretical analyses and simulation results demonstrate the superiority of the proposed algorithm in terms of achievable DOFs, estimation accuracy, spatial resolution and computational efficiency.

## Figures and Tables

**Table 1:** Steps for the proposed coprime array direction-of-arrival estimation algorithm.

**Step 1**: Derive coarray domain statistics based on the coprime array received signals x(l).
**Step 2**: Generate the coprime coarray covariance matrix ${\tilde{R}}_{yy}$ via Equation (13).
**Step 3**: Construct the signal subspaces of the shift invariant subarray pair $\Omega_{S,X}$ and $\Omega_{S,Y}$ via Equation (22).
**Step 4**: Obtain the rotational operator Ψ via Equation (28) based on the coarray domain rotational invariance.
**Step 5**: Calculate the DOA estimations via the closed-form solution in Equation (31).

**Table 2:** Computation time comparison of each algorithm.

	SSR [34]	SS-MUSIC [32]	Interpolation [37]	Proposed
500 Monte Carlo Trials	1089.71 s	9.48 s	352.43 s	1.52 s
Average Time	2.179 s	0.019 s	0.705 s	0.003 s

## Abbreviations

The following abbreviations are used in this manuscript:

CRB	Cramér–Rao Bound
DOA	Direction-of-Arrival
DOF	Degree-of-Freedom
ESPRIT	Estimation of Signal Parameters via Rotational Invariance Techniques
MUSIC	MUltiple SIgnal Classification
RMSE	Root Mean Square Error
SNR	Signal-to-Noise Ratio
SS-MUSIC	Spatial Smoothing MUSIC
SSR	Sparse Signal Reconstruction
ULA	Uniform Linear Array
